# Protective role of virgin coconut oil on potent biochemical biomarkers in Wistar rat model of comorbid depression

**DOI:** 10.5455/javar.2024.k794

**Published:** 2024-06-12

**Authors:** Chitra Pai Kulyadi, Anupama Noojibail, Nayanatara Arun Kumar, Sowndarya Kollampare, Prameela Manoor Dass

**Affiliations:** 1 Department of Radiology , University of Colorado, Anschutz Medical Campus , Aurora , Colorado, USA; 2 Department of Physiology , Kasturba Medical College Mangalore, Manipal Academy of Higher Education, Karnataka, Manipal , 576104, India.; 3 Department of Biochemistry , Kasturba Medical College Mangalore, Manipal Academy of Higher Education, Karnataka, Manipal , 576104, India; 4 Department of Anatomy , Kasturba Medical College Mangalore, Manipal Academy of Higher Education, Karnataka, Manipal , 576104, India

**Keywords:** Biochemical markers, chronic unpredictable stress, depression, virgin coconut oil

## Abstract

**Objective:**

Chronic stress arises from stressful situations in day-to-day life that are ignored or managed incorrectly. Long-term stress can have negative effects, especially when it plays a role in the development of neurological illnesses. Severe stress can also negatively impact emotional well-being. Virgin coconut oil (VCO) has numerous health advantages. The aim of this study was to assess how VCO affected the biochemical and behavioral characteristics of Wistar albino rats exposed to chronic, unpredictable stress.

**Materials and Methods:**

Healthy Wistar albino rats (150–200 gm) were split into two groups: experimental group and control group. Based on stress exposure and treatment with VCO and antidepressants, they were further divided into various subgroups. A chronic, unpredictable stress procedure was given for 21 days. After the experimental procedure, the rats were anesthetized, and through a cardiac puncture, blood was collected. The liver and brain were dissected to estimate different biochemical markers.

**Results:**

VCO proved to be a protective agent against chronic, unpredictable stress-induced changes in the biochemical parameters, hepatic enzyme activity, lipid profile, oxidative stress, and cognition.

**Conclusion:**

VCO might be helpful as an effective natural treatment that can be utilized to effectively combat chronic, unpredictable stress-induced changes in brain and liver tissue.

## Introduction

Any undesirable emotional experience linked with biochemical, physiological, and behavioral alterations has been referred to as stress [1]. Prolonged stress can have a deleterious effect on one’s health, leading to disturbances in normal homeostasis [2]. When compared to anticipated stressors, unpredictable stressors tend to have a higher negative impact on people. This is likely because unpredictable stressors are difficult to predict and involve uncertainty when events occur [3]. The chronic unpredictable stress (CUS) model is a very effective animal model for exploring the biological mechanisms leading to the pathogenesis of metabolic and endocrinal derangements. The free radicals involved in stress-induced oxidative damage lead to the onset of various disorders [4,5]. Serum cortisol level is one of the proven estimated biomarkers during stress exposure [6]. Balancing the changes in the basal glucocorticoid level is crucial for their beneficial and harmful effects. Serum lipid levels have been directly associated with cardiovascular health [7].

For many years, the food industry has relied heavily on coconut oil, and more recently, the use of virgin coconut oil (VCO) has increased [8]. VCO is procured using the traditional wet method of coconut oil extraction. The components present in the VCO have been well-linked to medicinal use [9,10]. This study attempts to explore the protective function of VCO in chronic stress-induced comorbid depression. The study focuses primarily on the powerful biochemical biomarkers in the CUS-induced depression rat model.

## Materials and Methods

### Ethical approval

The Institutional Animal Ethical Committee (IAEC) of Kasturba Medical College, Mangalore, has reviewed and approved the current experimental procedures (KMC/MNG/IAEC/30-09-2017). The Government of India’s Committee for Control and Supervision of Animal Experiments guidelines were strictly followed.

### Animals

The animals needed for the experimental procedures were procured from the Institutional Animal House. Healthy adult albino rats (2–3 months old) of the Wistar strain, weighing between 150 and 200 gm were selected. Each rat was kept in a cage and allowed to acclimate for a week in controlled light and temperature (25ºC ± 2ºC). They were fed with pellets and water.

### VCO

The Central Plantation Crops Research Institute, Kasaragod, Kerala, India, provided the VCO used in this investigation. VCO was made using the hot processing method [11] from the procedures and machineries developed at the ICAR-Central Plantation Crops Research Institute’s Agro Processing Complex in Kasaragod, Kerala, India.

### Drugs and chemicals

Arbour Assays^®^’ Detect X^®^ Corticosterone Enzyme Immunoassay Kit (Kit Catalogue Number K014-H5) was used to measure serum corticosterone [12]. A commercial kit from Diatek Healthcare Private Limited was used to estimate serum cholesterol, and the Drugs and Chemicals Cholesterol Oxidase-Peroxidase (CHOD-POD) method was used for analysis [13]. The blood glucose was checked by a glucometer [14].

### Experimental stress procedure

The experimental procedure for CUS was modified slightly from the previous studies [4,5,15]. The rats were made to swim against their will for sixty minutes on the first day. Rats were placed in a cage that was 45° slanted for the following hour. The rats were returned to their original cages after an hour of rest following each stress session. On the second day, the rats were put through an immobilization stress test. First, they were kept in a plexiglass restrainer, and then they were kept in the rotating chamber at 50 rpm. On day three, they were given a one-hour wet session in the cage, followed by swimming in cold water. The rats were made to swim in a cylindrical tank with a water height of 30 cm at 4°C–8°C during the cold swimming experiment. On the fourth day, six rats were housed in a cage for two hours to induce overcrowding stress, after which the rats received a foot shock (1.5 mA) for ten minutes. After five days, rats were kept in a dark room during the day and exploded with light during the night to alter the cycles of light and dark. On the sixth day, the rats received two types of stress treatments: a 30-min tail pinch stress, in which a pinch clip was applied 2 cm from the base of the tail, and each rat was kept in the cage covered with black paper for one hour as per the procedure of isolation stress. The rats were exposed to heat stress on day seven when they were placed in a hot air stream. This stress procedure also included overnight food and water restrictions. For three weeks, this seven-day stress procedure was carried out.

A total of 36 rats were used in this study. They were divided into six groups, each group having six animals (*n *= 6; *n* is the number of animals in each group). The control group of rats was not exposed to any stress. The stress group of rats was exposed to CUS for 21 days. The rats treated with VCO (2 ml/kg body weight; oral route) were grouped as the VCO group. The VCO group exposed to stress was named VCO+ Stress. The rats treated with the antidepressant (AD) drug imipramine (10 mg/kg body weight; oral route) and exposed to stress were included in the AD + stress group. Furthermore, the VCO + AD + stress group included the CUS rats treated with VCO and AD. After 21 days of stress, at the end of the experimental procedure, all the rats were anesthetized by injecting ketamine at a dose of (50 ml/kg body weight). Blood glucose levels were measured immediately by the tail-prick method using a glucometer device and reactive strips. For the serum lipid profile, the blood was collected by cardiac puncture and subjected to centrifugation. Thus, the obtained supernatant serum was used for the estimation of serum cholesterol. Harvesting of brain and liver tissue was done to determine the oxidative biomarkers.

### Preparation of tissue homogenates

Tissue homogenate was made at a ratio of 1 gm of tissue in 2 ml of phosphate buffer (0.2 M, pH 7.6). The supernatant obtained was centrifuged for 10 min at 40°C and used for biochemical analysis. Total protein was estimated using the Biuret method [16].

### Estimation of malondialdehyde (MDA)

MDA levels in the brain and liver were estimated by Kei Satoh’s method [17] with minor modifications. MDA was expressed as µmol/ml [18].

### Estimation of antioxidant GSH level

Using Ellaman’s reaction, reduced glutathione (GSH) was estimated [19]. GSH was expressed as µmol/g protein.

### Statistical analysis

Results were expressed as mean ± standard error mean. One-way ANOVA and post hoc tests were used. *p* < 0.05 was considered statistically significant.

## Results and Discussion

**Figure 1.**
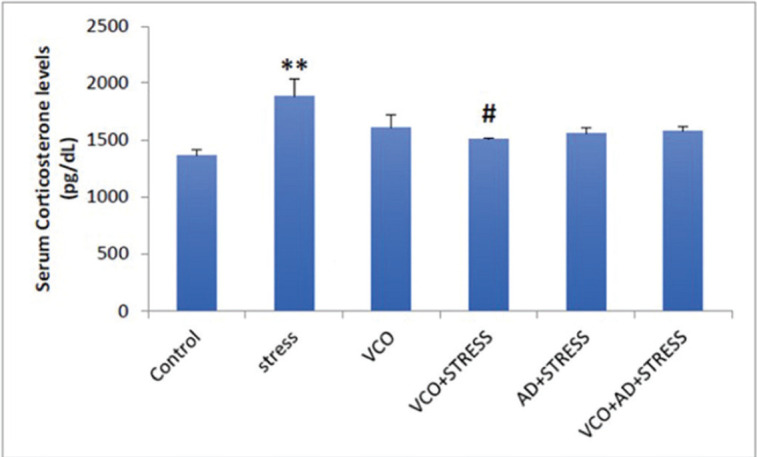
Serum corticosterone level in different experimental groups.

A comparison of levels of serum corticosterone among different groups is represented in Figure 1. CUS significantly increased serum corticosterone levels in comparison to the control group (*p* < 0.001). However, when stressed rats were treated with VCO, they showed a significant decline (*p* < 0.05) in serum corticosterone levels when compared to the rats exposed to CUS without treatment with VCO.

**Figure 2.**
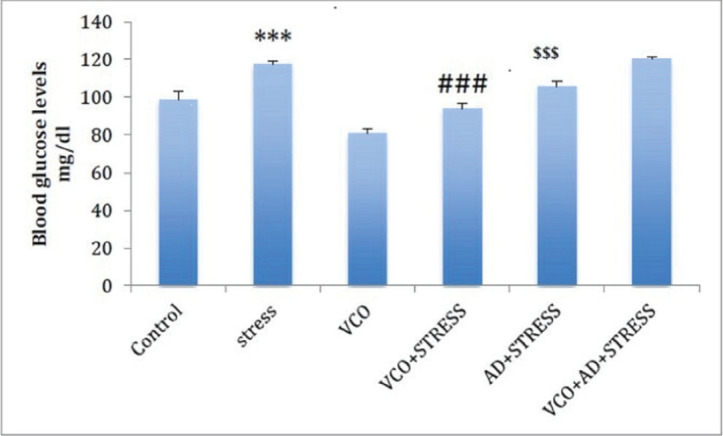
Blood glucose level in various experimental groups. ***p < 0.001; Control versus stress group, ^###^*p* < 0.001; Stress versus VCO+Stress, ^$$$^*p* < 0.001; Stress versus AD+Stress, ^@@^*p* < 0.01; Stress versus VCO +AD+Stress.

Figure 2 shows the comparison of levels of blood glucose between controls and different experimental groups. The stressed rats showed significantly increased blood glucose levels in comparison to control rats (*p* < 0.001). Treatment with VCO during the exposure to stress procedures showed a significant decline in baseline blood glucose levels as compared to controls (*p* < 0.001). In stressed rats treated with VCO and AD, there was a significant increase in (*p* < 0.001) blood glucose levels in comparison to both the VCO-treated stress group and the AD-treated stress group (*p* < 0.001).

A comparison of liver enzymes such as alanine transaminase (ALT), aspartate transaminase (AST), and alkaline phosphatase (ALP) between the control and experimental groups is shown in Table 1. In the stress-exposed group, there was a significant (*p* < 0.001) increase in levels of serum ALT and ALP compared to the control group. However, treatment with VCO in chronic stress-exposed rats caused a significant (*p* < 0.001) decline in the ALT and ALP levels. Stressed rats treated with both VCO and AD showed significantly low serum ALP in comparison to untreated stress rats (*p* < 0.001) and AD-treated stress rats (*p* < 0.001). A significant change in the levels of AST between the groups was not found.

Table 2 compares the levels of various lipid parameters among various groups. Exposure to stress significantly increased (*p* < 0.001) the serum cholesterol level as compared to the control group. The group treated with VCO during CUS showed a significant decline (*p* < 0.001) in the serum cholesterol level when compared to untreated stress rats. There was a significant (*p* < 0.05) rise in serum cholesterol in the animals treated with imipramine when compared to those treated with VCO. Exposure to stress did not show any significant (*p* < 0.001) changes in the serum triglyceride level. The triglyceride level significantly decreased (*p* < 0.01) in the AD-treated stress group. The VCO-treated group showed an increase in serum high-density lipoprotein (HDL) levels when compared to its respective control group, although this was not statistically significant.

The stress group showed significantly (*p* < 0.001) high levels of malondialdehyde in brain and liver tissues in comparison to control rats (Table 3). Treatment with VCO significantly decreased (*p* < 0.001) the MDA level in the liver and brain when compared to the untreated stress group. AD-treated stress rats also showed a significant decline (*p* < 0.001) in lipid peroxidation levels in comparison to the stress group. Stressed animals treated with both VCO and AD had significantly lower (*p* < 0.001) brain and liver malondialdehyde levels when compared to untreated stressed rats. A significant (*p* < 0.001) decrease in the levels of reduced GSH in the brain and liver tissue of stressed rats was seen when compared to controls. On treatment with VCO, the stressed group showed significantly (*p* < 0.05) increased GSH levels in brain and liver tissue. Treatment with both AD and VCO significantly (*p* < 0.001) reduced antioxidant levels in the brain in comparison to the VCO-treated stress group.

## Discussion

Stress is caused by negative emotions. Chronic stress disrupts the antioxidant balance, leading to tissue damage. Stress is the main cause of many diseases. In the present study, CUS was found to increase serum corticosterone. Hormonal secretion is a consequence of stress. Cortisol increases in acute stressful conditions and emotional outbreaks [20]. This adrenal hormone spike has been found to induce resistance to insulin hormone and favor hyperinsulinemia, thereby causing peaks in concentrations of plasma cholesterol and blood glucose [21]. Stressful circumstances might also elicit a hypothalamic-pituitary-adrenal (HPA) axis response. Chronic diseases can raise adrenal gland mass and activity, elevating cortisol metabolites in the bloodstream. Glucagon and adrenaline are stress hormones that boost hepatic production and breakdown of glucose and diminish muscular uptake, resulting in inadvertent hyperglycemia. However, in the present study, VCO could significantly restore corticosterone levels in chronic stress. VCO was also helpful in ameliorating blood glucose levels in stressed rats. This result reaffirms the conclusions of recent experimental studies and supports the claim that VCO possesses antistress and antihyperglycemic effects. The observed effect might be attributed to the associated components such as medium-chain fatty acids (MCFs), phenolic antioxidant compounds, and flavonoids preserved through novel production techniques.

**Table 1.** Comparison of serum liver enzymes among different experimental groups.

Groups	ALT (U/L)	AST (U/L)	ALP (U/L)
Controls	42.83 ± 4.28	118.83 ± 4.11	34.17 ± 4.91
Stress	71 ± 4^***^	153.5 ± 6.03	70 ± 4.33***
VCO	45.5 ± 3.73	111.5 ± 6.77	35.5 ± 3.1
VCO+stress	47.17 ± 3.68^##^	128 ± 8.65	40.17 ± 2.44^###^
AD+stress	77.83 ± 5.21^$$$^	167 ± 19.58	97.33 ± 3.94^$$$###^
Stress+AD+VCO	83.17 ± 2.59^$$$^	139.33 ± 7.6	49 ± 4.9^@@@ ##^

***p < 0.001; Control versus stress group, ###*p* < 0.001; Stress versus VCO+Stress, $$$*p* < 0.001; Stress versus AD+Stress, @@@*p* < 0.01; Stress versus VCO +AD+Stress.

**Table 2.** Comparison of serum lipid profile among different experimental groups.

Groups	Cholesterol	Triglycerides	HDL
Controls	43.17 ± 1.78	47.83 ± 3.68	76.67 ± 3.78
Stress	58.67 ± 3.06^**^	52.83 ± 2.51	61.17 ± 4.66^**^
VCO	38.83 ± 2.55	38.83 ± 2.55	87.17 ± 5.98
VCO+stress	40.33 ± 1.02^###^	53.67 ± 6.55	74.83 ± 4.78
Stress+AD	51.33 ± 3.46^$^	110.17 ± 5.29^###$$$^	65.83 ± 2.7
VCO+STRESS+AD	49.33 ± 2.25	101 ± 4.73	73.17 ± 4.24

**p < 0.001; Control versus stress group, ^###^* p* < 0.001; Stress versus VCO+Stress, $* p* < 0.05, $$$* p* < 0.001; Stress versus AD+Stress.

**Table 3.** Changes in MDA and GSH levels in the control group and experimental groups.

Groups	MDA levels in brain (µmoles)	MDA levels in liver (µmoles)	GSH levels in brain (µmoles/g protein)	GSH levels in liver (µmoles/gm protein)
Control	0.21 ± 0.005	0.18 ± 0.013	1.15 ± 0.019	0.370 ± 0.001
Stress	0.47 ± 0.04^***^	0.45 ± 0.034^***^	0.39 ± .043^***^	0.12 ± 0.003^***^
VCO	0.19 ± 0.007	0.15 ± 0.002	1.19 ± 0.166	0.38 ± 0.027
VCO+stress	0.30 ± 0.03^###^	0.29 ± 0.024^###^	0.820 ± 068^##^	0.22 ± 0.01^###^
AD+stress	0.23 ± 0.02^###^	0.22 ± 0.002^###^	0.16 ± .009^$$$^	0.24 ± 0.003^###^
VCO+AD+stress	0.20 ± 0.003^##^	0.12 ± 0.002^###$$^	0.19 ± 0.009^$$$^	0.26 ± 0.002^###^

***p < 0.001; Control versus stress group, ^##^*p* < 0.01, ^###^*p* < 0.001; Stress versus VCO+Stress, ^$$^*p* < 0.01, ^$$$^*p* < 0.001; Stress versus AD+Stress.

In the present experiment, exposure to CUS showed a significant rise in blood glucose concentrations. Stress hormones such as glucagon and adrenaline increase hepatic glycogenolysis and gluconeogenesis, reducing muscle glucose uptake and resulting in hyperglycemia. Psychotic stress is a major cause of elevated blood glucose levels. In the current study, compared to untreated stress rats, supplementation with VCO in chronic stress significantly reduced blood glucose levels and total cholesterol. Stress-induced increases in glucocorticoids cause a variety of physiological changes that help both people and animals react appropriately to stressful situations. Glucocorticoids play a substantial role in regulating the supply and demand of energy in animals by increasing blood sugar levels and promoting triglyceride catabolism in adipose cells. MCF increases the transport of triglycerides and increases their catabolism, which improves absorption from the intestinal tract into the portal circulation for easy metabolism and excretion into bile. This mechanism effectively and efficiently uses triglycerides to block the synthesis and transport of cholesterol [22]. The potent antioxidants, flavonoids, and phenolic acids present in the VCO might be linked to antistress activity and an antihyperglycemic effect.

In the current study, the liver enzyme levels in CUS were raised significantly, indicating CUS-induced depression and liver injury. Chronic stress-induced depression might injure hepatocytes, with a dire shift in the metabolism of phospholipids, synthesis of bile acid, and apoptosis of hepatocytes [23]. The present observations are in confirmation of the previous report [23]. However, the administration of VCO to the stressed rats was successful in mitigating the ALT and ALP levels. In the present study, exposure to two unpredictable stressors a day for 21 consecutive days resulted in oxidative damage in the brain and liver tissues, as determined by, increased levels of MDA and decreased GSH. Brain cells use more oxygen, and they are vulnerable to damage from free radicals. Previous reports also suggest a stress-induced increase in MDA levels in the various regions of the brain tissues, which include the cerebral cortex, midbrain, hippocampus, and cerebellum [24]. GSH is essential for the brain’s detoxification of reactive species. Stress raises ROS levels while lowering GSH levels [25]. The marked depletion of brain GSH, which is protective against oxidative damage, might have caused the enhancement of lipid peroxidation [25].

The results of the current study agree with those of earlier research, which confirms that stress exposure damages the liver, aggravating hepatic fibrosis and liver cirrhosis [26]. Polyphenols found in VCO might raise the levels of antioxidants, decreasing lipid peroxidation and inflammation [27]. The majority of the physiologically active primary components, such as polyphenols and tocopherols, which have strong antioxidant qualities, are retained in VCO, which has been extracted through wet processing. This could have a significant impact on lipid peroxidation prevention. Prior research indicates that VCO’s polyphenols were more effective than those of coconut oil and ground nut oil at preventing lipid peroxidation in microsomes and in vitro LDL oxidation [24–26]. Prolonged stress worsens the effects of oxidative pathways that increase the production of oxygen free radicals. According to current research, oxidative stress in vital organs like the liver and brain increases with exposure to CUS. This is in accordance with the previously published findings [28,29]. Furthermore, VCO repaired the damage that stress had caused to these organs. This suggests that VCO may have protective effects against the harmful effects of chronic stress on the liver and brain. The present study did not include specific areas of the brain to determine the impacts of oxidative damage, and histological aspects have not been considered. However, further molecular investigations are necessary to conclusively confirm and provide more evidence regarding the effects of VCO.

## Conclusion

VCO might have improved the antioxidant system in the liver and brain by decreasing oxidative stress. This may therefore prove to be a potential AD because it has a protective effect on liver physiology and brain dysfunction, lowering anxiety levels. This study proves the protective effect of VCO on the endocrine and metabolic responses of Wistar rats to chronic, unpredictable stressors. This is proved by the effective glucocorticoid responses, as evidenced by a decrease in serum corticosterone levels, and an improvement in lipid metabolism, as demonstrated by lower blood glucose and serum cholesterol levels. As a result, VCO might prove to be a useful metabolic enhancer under long-term stress. VCO could potentially modulate the pathways associated with oxidative stress and reinforce the antioxidant system in the brain. The organic substances present in the VCO, such as squalenes, tocopherols (vitamin E), polyphenols, and sterols, could be related to their potent antioxidant actions. Based on the observed protective role of VCO, it could be suggested as a novel natural treatment for depression.

## List of Abbreviations

AD, Antidepressants; CUS, Chronic unpredictable stress; MDA, Malondialdehyde, MFA, Median Chain Fatty acids; VCO, Virgin coconut oil.
